# An African‐specific ABCA7 Frameshift Deletion associated with Alzheimer's Disease alters Lipid Metabolism in neurons

**DOI:** 10.1002/alz70855_104992

**Published:** 2025-12-24

**Authors:** Younji Nam, Brooke A. DeRosa, Charles G. Golightly, Aura M Ramirez, Patrice G Whitehead, Larry D Adams, Takiyah D. Starks, Michael L Cuccaro, Allison M Caban‐Holt, Jonathan L Haines, Biniyam A. Ayele, Goldie S Byrd, Farid Rajabli, Derek M. Dykxhoorn, Juan I Young, Jeffery M Vance, Margaret Pericak‐Vance

**Affiliations:** ^1^ John P. Hussman Institute for Human Genomics, University of Miami Miller School of Medicine, Miami, FL, USA; ^2^ University of Miami Miller School of Medicine, Miami, FL, USA; ^3^ Dr. John T. Macdonald Foundation Department of Human Genetics, University of Miami Miller School of Medicine, Miami, FL, USA; ^4^ Wake Forest University School of Medicine, Winston‐Salem, NC, USA; ^5^ School of Medicine, Case Western Reserve University, Cleveland, OH, USA; ^6^ John P. Hussman Institute for Human Genomics, University of Miami Miller School of Medicine, Miami, FL, USA, Miami, FL, USA; ^7^ Addis Ababa University, Addis Ababa, Addis Ababa, Ethiopia

## Abstract

**Background:**

ATP‐binding cassette sub‐family A member 7 (*ABCA7*) is a lipid transporter across cellular membranes and has its highest expression in neurons. A 44‐base pair deletion in the gene is significantly associated with Alzheimer disease (AD) in African Americans (AA) with epidemiological data suggesting it interacts with APOE3/4 heterozygotes to lower the age‐of‐onset. This deletion produces a truncated protein (p.Arg578Alafs). To determine the impact of this deletion on lipid metabolism, cortical neurons derived from isogenic pairs of induced pluripotent stem cells (iPSCs) that either lack or have the deletion were created and characterized.

**Methods:**

A FLAG tagged *ABCA7* gene with and without the deletion was transfected into HEK cells to analyze the stability and localization of the truncated version of ABCA7. Isogenic iPSC lines from three cognitively intact AA individuals into which the *ABCA7* deletion was introduced were differentiated into neurons. Lipid accumulation was induced in the transfected HepG2 cells and isogenic neurons through the application of Oleic Acid and subsequent lipid droplet levels were assessed. Further, RNAseq and lipidomics was conducted on the isogenic neurons.

**Results:**

The truncated *ABCA7*‐tagged protein was stable and localized to the plasma membrane in transfected HEK cells, compared to that seen with wild‐type protein. The overexpression of the *ABCA7‐*44bp deletion in HepG2 cells led to a higher accumulation of lipid droplets. Similarly, the isogenic homozygous *ABCA7* deletion neurons showed significant increased numbers of lipid droplet. Transcriptome analysis showed that the *ABCA7* transcripts were expressed at similarly low levels in all isogenic neurons. Lipidomics data showed that the isogenic homozygous *ABCA7* deleted neurons have a >1.2‐fold cellular increase in phosphocholine and phosphoethanolamine compared to their isogenic controls. This is interesting in the light that it is known that the relative abundance of phosphocholine and phosphoethanolamine regulates the size and dynamics of lipid droplets.

**Conclusions:**

Our data suggest that the AA‐specific deletion in *ABCA7* produces a membrane localized truncated protein that alters lipid metabolism within neurons. Studies into why some individuals appear to escape the effect of this variant may highlight compensatory/protective mechanism that could be used for therapeutic development for all ancestries and populations.

